# Circulatory Insufficiency and Hypotension Related to the Ductus Arteriosus in Neonates

**DOI:** 10.3389/fped.2018.00062

**Published:** 2018-03-15

**Authors:** Danielle R. Rios, Soume Bhattacharya, Philip T. Levy, Patrick J. McNamara

**Affiliations:** ^1^Section of Neonatology, Department of Pediatrics, Texas Children’s Hospital, Baylor College of Medicine, Houston, TX, United States; ^2^Division of Neonatology, Department of Paediatrics, Western University, London, ON, Canada; ^3^Division of Newborn Medicine, Department of Pediatrics, Washington University School of Medicine, Saint Louis, MI, United States; ^4^Division of Neonatology, Department of Paediatrics and Physiology, University of Toronto, Toronto, ON, Canada

**Keywords:** ductus arteriosus, hypotension, hemodynamics, echocardiography, shunt volume

## Abstract

The biological role of the ductus arteriosus (DA) in neonates varies from an innocent bystander role during normal postnatal transition, to a supportive role when there is compromise to either systemic or pulmonary blood flow, to a pathological state in the presence of hemodynamically significant systemic to pulmonary shunts, as occurs in low birth weight infants. Among a wide array of clinical manifestations arising due to the ductal entity, systemic circulatory insufficiency and hypotension are of significant concern as they are particularly challenging to manage. An understanding of the physiologic interplay between the DA and the circulatory system is the key to developing appropriate targeted therapeutic strategies. In this review, we discuss the relationship of systemic hypotension to the DA, emphasizing the importance of critical thinking and a precise individual approach to intensive care support. We particularly focus on the variable states of hypotension arising directly due to a hemodynamically significant DA or seen in the period following successful surgical ligation. In addition, we explore the mechanistic contributions of the ductus to circulatory insufficiency that may manifest during the transitional period, states of maladapted transition (such as acute pulmonary hypertension of the newborn), and congenital heart disease (both ductal dependent and non-ductal dependent lesions). Understanding the dynamic modulator role of the ductus according to the ambient physiology enables a more precise approach to management. We review the pathophysiology, clinical manifestations, diagnosis, monitoring, and therapeutic intervention for the spectrum of DA-related circulatory compromise.

## Introduction

**Figure 1:**
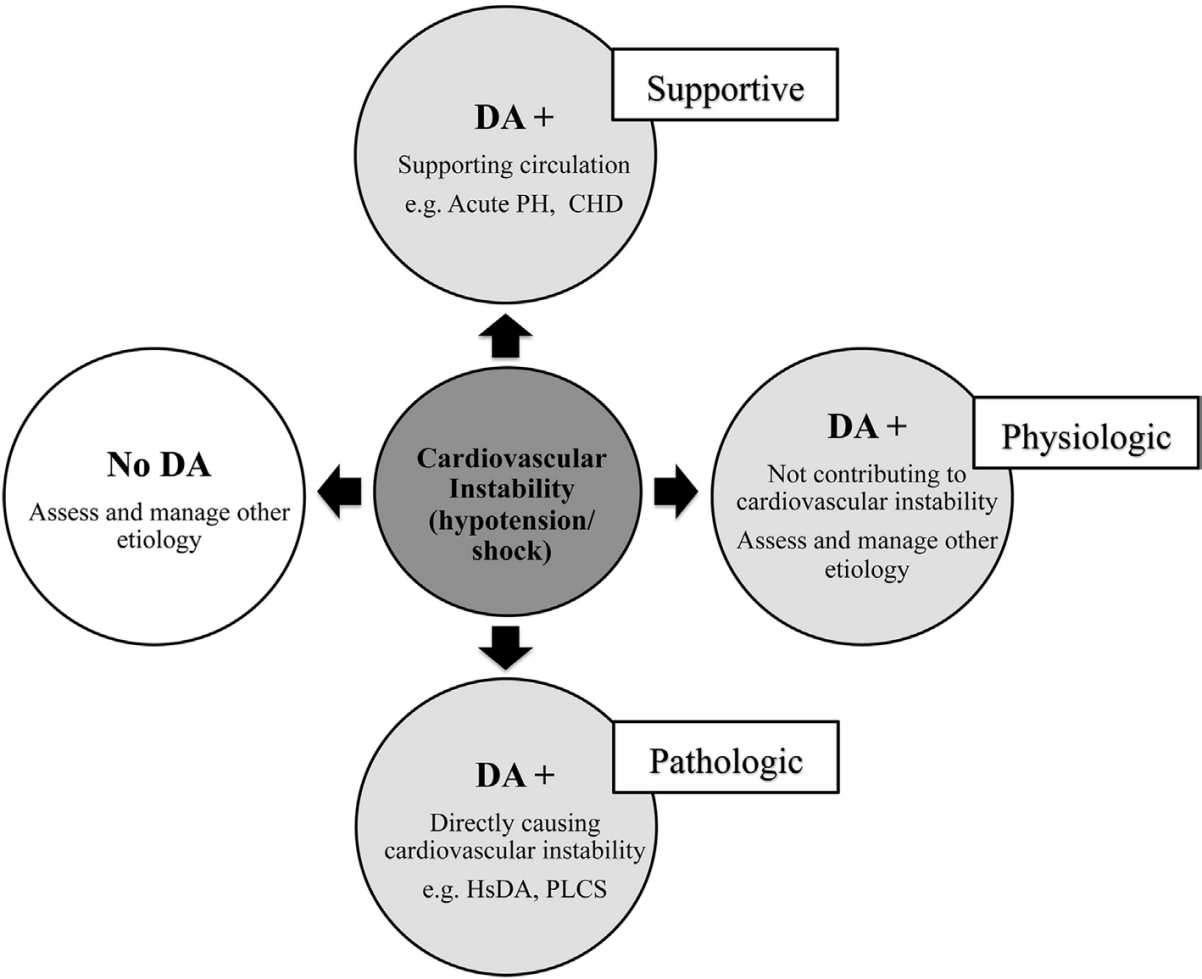
Relationship between circulatory instability and the ductus arteriosus (DA) in neonates. Acute PH, acute pulmonary hypertension of newborn; CHD, congenital heart disease; hsDA, hemodynamic significant DA; PLCS, post-ligation cardiac syndrome.

The ductus arteriosus (DA) has a multifaceted biological role in the context of hemodynamic instability in neonates that ranges from physiologic and supportive to pathologic (Figure 1) (1). Found in (almost) every infant at birth, the DA is an anatomical structure that is crucial in maintaining fetal circulation and usually undergoes spontaneous closure after aiding a normal circulatory transition. A patent DA (PDA) is a DA that remains open past the natural history. The hemodynamically significant DA (hsDA) represents the hemodynamic situation where the DA not only remains patent, but where the volume of the transductal shunt is sufficiently large that cardiovascular compromise may ensue. In addition, hypotension and circulatory insufficiency are also well-known complications following treatment to close a DA (2). In this review, we present a novel method to assess hemodynamic instability associated with the DA and discuss a physiology-based approach to the diagnosis and management. The complexity of the physiology of these hypotensive states is such that there are clinical situations where the patency of the DA can favorably modulate and support circulation to vital organs. Differentiation of the supportive from the pathological role of the DA is, therefore, essential. Our aim is to present the complex mechanistic interplay between hypotension and the DA that will guide decision-making in critically ill neonates.

## DA in Fetal Circulation and Early Transition: Revisiting the Normal Physiology

At birth, infants must navigate a series of complex adaptive sequences to successfully transition from fetal to postnatal circulation. The placenta serves as the organ of gas exchange as the lungs only receive a small amount of blood flow (3, 4). In the fetal circulation, oxygenated blood from the placenta is diverted away from the right heart and travels to the left heart *via* the foramen ovale (5). The left heart distributes the oxygenated blood to the brain, myocardium, and peripheral circulation, and the right heart [which ejects about 65% of the combined ventricular output (6, 7)] receives deoxygenated blood from the fetal veins and ejects it into the pulmonary artery (4). Between 40 and 55% of the combined cardiac output (CO) diverts through the DA away from “the high-resistance pulmonary vascular bed to the low-resistance umbilical-placental circulation” (4, 7). Therefore, in the fetal circulation, the DA has the crucial biological role of maintaining a right-to-left shunt. The elevated pulmonary vascular resistance (PVR) that results from fluid-filled fetal alveoli helps drive this shunt, while the fetal hypoxic environment promotes production of endogenous prostaglandins, primarily prostaglandin E2 (PGE_2_) and prostaglandin I_2_ (PGI_2_), within the lumen of the DA to ensure its ongoing patency. In addition, the intricate balance between the maternal uterine and fetal placental prostaglandin pathway expression profiles also drives the shunt direction.

After birth, the first breath of relatively oxygenated air results in lung expansion and clearance of fetal lung fluid. Mechanical stretch of the newly inflated lungs and relief of alveolar hypoxia dramatically decrease PVR, leading to an increase in pulmonary blood flow (PBF) (8). Clamping of the umbilical cord removes the low-resistance placental flow with a subsequent rise in the systemic vascular resistance (SVR) and reduction in right heart preload. Both cold stress and catecholamine surges will further cause the SVR to increase (9). As PVR decreases, the direction of flow across the DA changes from all right-to-left (as is seen with fetal circulation) to bidirectional. With continued decrease in PVR, the flow pattern becomes predominantly left-to-right and directs blood from the aorta to the pulmonary arteries (10). Such a change in the shunt direction results in a small, but significant, increase in PBF volume that increases pulmonary venous return and leads to higher left atrial pressures. This causes displacement of the flap of the foramen ovale over the rims of the fossa (5) and stops the right-to-left flow through it. Residual left-to-right flow through the foramen ovale can commonly be seen for a few days as the circulation readjusts.

As the lungs become the organ of gas exchange, the postnatal increase in PBF leads to a rapid rise in lung perfusion and elevation of neonatal oxygen saturation. During the physiologic transition, the increase in systemic arterial oxygen tension and decrease in circulating prostaglandin E levels (including placental prostaglandins) promotes the constrictive effect of oxygen on the ductal tissue (6). This constriction results in *functional* closure of the DA within the first 10–15 h after birth. In healthy infants, the functional closure triggers the subsequent process of *anatomical* closure that begins at the pulmonary end (11) and is often completed within 2–3 weeks of life. In some term infants and many preterm infants, the process of physiologic ductal closure can be maladaptive, delayed, or even arrested. The DA will close spontaneously in most term and preterm infants. In a select number of preterm infants, medical therapy will be required to augment closure of the DA if it is considered hemodynamically significant; an even smaller proportion might benefit from surgical ligation. Despite closure (either spontaneously or with medical therapy), the DA may reopen with changes in the hemodynamic milieu (12), and the persistence of the DA can eventually have serious hemodynamic consequences.

## Assessment of DA-Related Hemodynamic Instability: A Novel Approach

The clinical significance of systemic hypotension must be carefully interpreted based on the variance in biologic role of the DA in different physiologic phases and disease states (5, 10). This will enable a clearer understanding of how hemodynamic consequences evolve in the context of a DA and ensure proper diagnosis, monitoring, and therapeutic intervention when necessary. Although hemodynamic assessment of systemic perfusion with echocardiography is more physiologically informative, our clinical suspicion of cardiovascular instability should precede the use of echocardiography. This crucial “first step” of clinically identifying systemic hypotension with low systemic blood flow (SBF), however, along the spectrum of DA-related circulatory dysfunction, may be difficult based on the inherent limitations of its definition. Mean arterial pressure (MAP) is the most common clinical parameter used to define hemodynamic instability and is often used as a surrogate of end-organ perfusion, where clinicians rely on the “absolute value” to guide intervention (13). The lack of comprehensive normative datasets in neonates (13), disconnect between MAP, CO, and target organ flow (10), and overreliance on a singular MAP rather than systolic and diastolic blood pressures separately (14), limit the use of MAP to monitor and treat low blood flow states in preterm and term infants (10).

In clinical terms, neonatologists generally use one of the following three thresholds (5) to define systemic hypotension based on blood pressure; first, MAP less than gestational age (GA, in weeks) generated by consensus opinion (15) derived from (i) the notion that the majority of “healthy” preterm infants have an MAP greater than or equal to their GA and (ii) an observation from one normative dataset that GA roughly correlated with the 10th percentile for MAP (16); second, MAP less than 30 mmHg based on the assumption that cerebral blood flow becomes pressure dependent at a MAP around 30 mmHg with loss of cerebral autoregulation and cerebral white matter damage (17, 18), and a population study, which found that the 10th percentile for infants of all GA is at or above 30 mmHg at day 3 of life (19); and finally, systolic arterial pressure (SAP) and diastolic arterial pressures (DAPs) less than the third percentile according to GA based on normative data (10, 20, 21).

Since MAP rises as gestational and postnatal age increase (22), a single cut-off threshold of MAP-based hypotension (either gestational age or number specific) may not be plausible as it varies across gestation in health and disease (5). Furthermore, the relative lack of a clear threshold below which autoregulation and target organ perfusion are compromised limits the applicability of such definitions (5). Owing to the importance of CO (rather than MAP) in the regulation of cerebral blood flow (23, 24), and the recognition of multiple confounding physiologic differences that alter how MAP interacts with SBF, studies compared MAP with superior vena cava (SVC) flow, an estimate of CO, and reported a weak correlation between these two measures (25–27). One study even found that preterm infants with reduced SVC flow had normal or high MAP in the first day of life (28). Although there is a lack of data demonstrating any benefit to instituting treatment to correct MAP above these thresholds (5), using a MAP <30 mmHg, when compared to a MAP < GA, as a clinical parameter to predict low CO (low SVC flow) resulted in an increase in sensitivity from 30 to 59%, but at the expense of a decrease in specificity from 88 to 77% (25).

**Figure 2:**
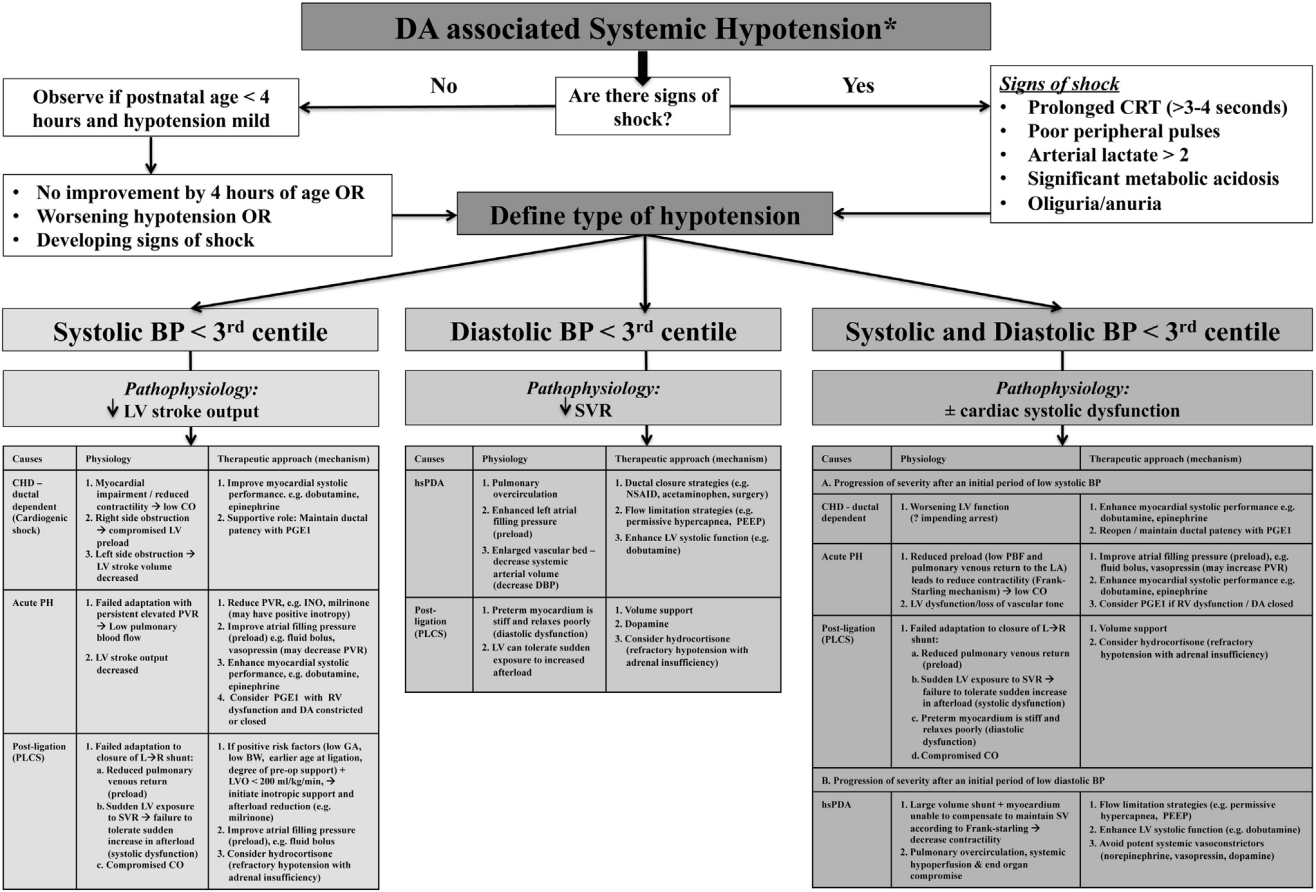
Algorithm for the assessment and treatment of ductus arteriosus (DA)-associated hypotension according to systolic, diastolic, and combined systolic and diastolic categories. *Global assessment of systemic perfusion from integration with arterial pressure thresholds may provide early clinical insights regarding pathophysiologic determinants of hemodynamic instability, but ascertainment and confirmation of the presence of hemodynamics significance must be obtained using comprehensive echocardiography.

The individual components of MAP, SAP, and DAP may provide more valuable insight into the overall wellbeing of the cardiovascular system as compared to MAP alone and may facilitate enhanced diagnostic consideration regarding specific causality (5, 10). SAP represents the force of blood exerted on the arterial wall in systole and reflects left ventricle (LV) contractile force and effective CO; therefore, a low value may indicate reduced stoke volume (which is influenced by preload, contractility, and afterload). DAP represents the resting pressure of blood on the vessels and is reflective of systemic vascular tone/resistance and intravascular blood volume status (10). Considering MAP as two separate components (SAP and DAP) will reflect a more physiologic basis for diagnosis, treatment, and monitoring of low blood flow states (5). For example, in the setting of the hsDA, identifying initial diastolic hypotension with preserved systolic pressure (wide pulse pressure) reflects the initial stages where right ventricle (RV) afterload declines and SVR rises after birth in the face of mild systemic end organ dysfunction. A progressive decline in SAP may reflect a situation where the LV cannot augment systolic performance to compensate for a rapid increase in preload (high volume of PBF returning to left atrium) leading to suboptimal CO with both systolic and diastolic hypotension and severe end organ dysfunction. This contrasts with states where SAP is low initially but in the setting of normal/high DAP; for example, following ligation of PDA, acute pulmonary hypertension of the newborn (acute PH), and select ductal-dependent congenital heart lesions will drive different approaches to optimize CO. Where echocardiography assessment of CO is not feasible, surrogate measures (e.g., urinary output, arterial pH, and lactate) should be used to appraise the impact of arterial pressure thresholds on cellular homeostasis. Loss of vascular tone and ventricular dysfunction will lead to diastolic hypotension with advanced disease. Combined low SAP and DAP may reflect a “common end-point” that occurs when the cardiovascular system fails to adapt to ongoing hemodynamic stress (10). Comprehension of the pathophysiology of hemodynamic instability (whether low DAP, low SAP, or both diastolic and systolic hypotension) in relation to the DA in these conditions will guide management decisions, but its relevance to neonatal outcomes still needs prospective evaluation (Figure 2) (5, 10). Arterial pressure does not predict CO, and although it may provide insight regarding the possibility of an hsDA, ascertainment of the presence of hemodynamics significance can only be obtained using comprehensive echocardiography. Global assessment of systemic perfusion and integration with arterial pressure thresholds may provide early clinical insights regarding pathophysiologic determinants of hemodynamic instability, which may be further enhanced by comprehensive echocardiography leading to medical precision.

## Hypotension Resulting from AN hsDA

An hsDA in the premature neonate can result in a wide spectrum of complex circulatory consequences that manifest with clinical signs of pulmonary overcirculation or systemic hypoperfusion as the volume of left ventricular output (LVO) is increasingly diverted from the systemic to the pulmonary circulation. The magnitude of transductal blood flow is modulated by the specific sub-components of Poiseulle’s law namely transductal pressure gradients, diameter, length, and viscosity. There are multiple mechanistic pathways that lead to the circulatory imbalance with an hsDA that can be characterized by the presence of diastolic and/or systolic hypotension (5, 10). The risk of refractory hypotension is considerably increased in preterm neonates with an hsDA (29), but in order to define systemic hypotension in the context of an hsDA, appraisal of the link between MAP and CO must account for SVR, according to the relationship, mean blood pressure − right atrial pressure = CO × SVR (5). As PVR declines after birth, there is a progressive development of a left-to-right transductal shunt that can evolve through stages of sufficient to inadequate compensatory rise in CO in the setting of an hsDA.

In the initial stage of systemic hypotension related to the hsDA, the LV myocardium adapts to increasing left atrial preload (from the increased pulmonary venous return) by increasing its stroke volume (SV). The hsDA will enlarge the vascular bed and lower net SVR, further augmenting the SV with a reduction in LV afterload. Clinically, this results in the “paradox” of high ventricular performance with increased pre-ductal CO (10, 30–32), characterized by low DAP with preserved SAP (wide pulse pressures), but post-ductal end organ compromise (33). Low coronary perfusion in the setting of critical low aortic diastolic pressure may also be contributory (34, 35). The inherent functional dysmaturity that characterizes the preterm myocardium may further impair these compensatory mechanisms.

In general, the premature myocardium is more sensitive to changes in afterload with an ineffective contractile mechanism and a steeper slope on the stress–velocity curve (10). In addition, the preterm myocardium is also predisposed to diastolic dysfunction owing to the presence of high non-contractile and non-compliant collagen fibers (36). Depending on the degree of myocardial immaturity, neonates with an hsDA may fail to adapt to the sudden increase in preload on the left atrium and properly augment its systolic performance (5, 10). With more advanced disease, the LV will be unable to maintain the SV according to the Frank–Starling relationship, and the inherent decreased contractility will lead to further pulmonary congestion, systemic hypoperfusion, and severe end organ compromise. This second phenotype of low SVR and suboptimal CO (low SV) will lead to an overall decline in both SAP and DAP (10). The presence of a large left–right transatrial shunt may also limit augmentation of CO with similar consequences.

The “ductal steal phenomenon” is another major contributor to circulatory imbalance. In the presence of a high systemic to pulmonary pressure gradient, an hsDA allows a high volume left-to-right shunt to “steal” blood away from the systemic circulation and into the pulmonary bed. This progressive steal phenomenon will lead to systemic circulatory insufficiency that can further compromise end organ perfusion, particularly in the postductal organs such as kidneys and gastrointestinal tract (30, 31, 37–40). Even pre-ductal organs, e.g., heart and brain, are not spared from the effects of diastolic runoff as large volume ductal shunts can lead to a coronary steal phenomenon (34, 35) and cerebral hypoperfusion secondary to impaired perfusion pressure. The immature cerebral autoregulation in the premature neonate makes this at-risk population particularly vulnerable to the effects of low CO and ductal steal, thereby increasing the risk of white matter injury (41). Additionally, the risk of intraventricular hemorrhage (IVH) proportionately increases when the cerebral perfusion is affected by an hsDA (42, 43), as infants who developed IVH were noted to have a period of sustained augmentation of pre-ductal CO just prior to the development of IVH (43).

The presence of diastolic and/or systolic hypotension can provide insight into the degree of hemodynamic significance of the PDA; however, this approach is yet to be assessed systematically (5). Diagnosing the exact cause of circulatory instability in neonates and ascribing it to an hsDA requires careful synthesis of clinical clues, laboratory parameters, and echocardiographic findings. Several PDA severity risk scores that evaluate hemodynamics and clinical significance, predominantly based on echocardiographic parameters, do exist (44–49). These scores provide an indirect estimate of shunt volume and the magnitude of impact to vital organs, essential for optimization of management. Clinical integration of echocardiography parameter with arterial pressure thresholds cannot be over emphasized, especially in situations where septic physiology co-exists with a large hsDA. Newer modalities of comprehensive hemodynamic monitoring and target organ flow, e.g., near infrared spectroscopy, non-invasive CO monitoring, and amplitude integrated electro encephalogram may also provide useful additive information (50). A complete hemodynamic monitoring protocol that can combine the real-time information from all these tools is an attractive idea that may facilitate early diagnosis and guide treatment, and is currently under investigation (51, 52).

The principles of management may also differ based on the presence of diastolic and/or systolic hypotension. In the initial phase of an hsDA, the first line management of diastolic hypotension revolves around augmentation of ductal closure with shunt limitation strategies that decrease the left-to-right flow gradient by increasing PVR [e.g., permissive hypercapnemia, minimizing FiO_2_ requirement (53), optimizing positive end expiratory pressure (54), and promoting tissue oxygen delivery with higher hematocrit levels (55)]. These non-invasive, non-pharmacologic strategies may lead to a resolution of the low diastolic pressure, improve CO and SBF, by limiting pulmonary overcirculation, and avoidance of the need for medical intervention. Such shunt limiting strategies may also act as temporizing measures while definitive medical treatment for PDA is underway (53, 54). Limitation of shunt volume during administration of medical treatment may enhance PDA closure, although the latter has not been formally studied. Despite augmentation of both non-pharmacologic and medical therapies, selected infants may still progress to a more advanced disease state with cardiovascular compromise characterized by both diastolic and systolic hypotension. These infants have persistent systemic hypoperfusion (decreased LVO) necessitating the addition of positive inotropes to support systolic dysfunction without causing systemic vasoconstriction. Dobutamine, with its ability to raise CO by increasing SV and preserving SVR through offsetting β2-mediated vasodilation and α1-mediated vasoconstriction in the peripheral circulation, may be the preferred treatment option to support systolic dysfunction with an hsDA. In contrast, dopamine, acting as an agonist of multiple receptors, including α, β, and dopaminergic receptor, raises SV, PVR (56, 57), and SVR (10, 58) in the setting of the hsDA. Although arterial pressure may be augmented with dopamine, the negative impact of profound vasoconstriction on end-organ perfusion is a concern as it may actually be hidden from clinical detection (59). Other potent systemic vasoconstrictors, such as vasopressin and norepinephrine, should also be avoided for their similar effects on systemic vascular tone. A comparative evaluation of these agents in the setting of hsDA has not been performed (10).

## Hemodynamic Instability Following PDA Treatment

Characteristic hemodynamic changes of circulatory insufficiency and systemic hypotension can often be seen in the period following closure of the PDA. Awareness of the potential hemodynamic consequences of ductal treatment and understanding the pathophysiologic basis of these alterations are key to effective management of neonates in the posttreatment period. Indomethacin, often used to augment pharmacological PDA closure, can lead to significant reduction of cerebral, mesenteric, renal, and even coronary blood flow velocities due to its potent vasoconstrictor properties (38, 60–62). Though such effects are temporary and revert back to normal once the treatment is completed, they still have the potential to lead to serious consequences related to target organ flow, e.g., necrotizing enterocolitis, renal dysfunction, etc. Agents such as ibuprofen and acetaminophen have not demonstrated such deleterious effects on organ perfusion and are increasingly being preferred by clinicians over indomethacin for treatment of an hsDA (1, 63). There remains a gap of knowledge on ibuprofen prophylaxis and treatment side-effect profiles in preterm infants, highlighting the need for further randomized prospective studies to isolate the impact of PDA and its treatment options on the risks in this population (64).

Cardiovascular instability in the postoperative period is a serious concern after PDA ligation surgery (65, 66). This entity has been described as the post-ligation cardiac syndrome (PLCS) and occurs secondary to sudden, drastic changes of the loading condition on the left side of the heart following ligation. The clinical phenotype of PLCS is often presented by 6–12 h post-ligation and characterized by systolic hypotension (less than third percentile) and normal or elevated diastolic BP that often requires inotropic support (66–69). Concomitant to the systemic circulatory insufficiency, PLCS (left untreated) can present with difficulty in ventilation and increasing oxygen demands from left heart failure and the resultant pulmonary congestion and pulmonary venous hypertension. Risk factors associated with PLCS include earlier age at ligation (69), lower birth weight (70), younger gestational age (70–72), large PDA (65), and level of preoperative cardiorespiratory support (66, 68, 72). Recent studies have demonstrated little to no clinical PLCS with catheter-based closure interventions, but the infants were older and weighed more at the time of closure compared to studies where infants underwent surgical ligation (73). Although there was still echocardiographic evidence for diminished left ventricular systolic function with the catheter-based approach (73), larger scale prospective studies are needed to determine the true incidence of PLCS with this procedure.

**Figure 3:**
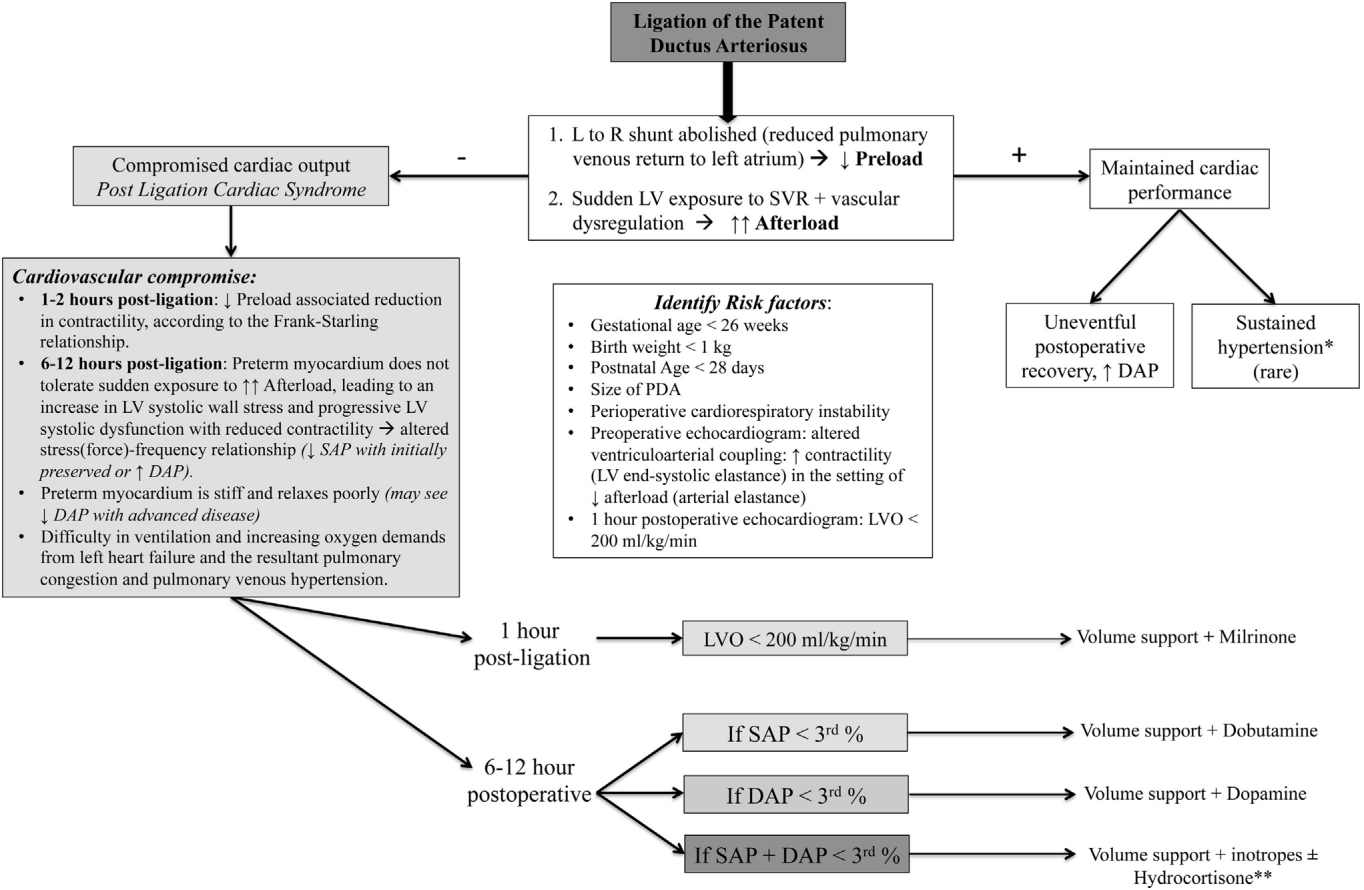
Hemodynamic alterations and clinical algorithm following patent DA (PDA) ligation. L to R, left to right; GA, gestational age; SAP, systolic arterial pressure; DAP, diastolic arterial pressure; PLCS, post-ligation cardiac syndrome. *Transient hypertension, with a predominant increase in diastolic blood pressure, has also been observed in the immediate postoperative period following PDA ligation (75), lasting for a variable amount of time, but rarely beyond the first 24–48 h post-ligation. Persistent hypertension lasting days or weeks and hypertension needing treatment are relatively rare complications, with only a few cases reported in infants and older children (76, 77). Post-PDA treatment hypertension is ascribed to the increased systemic vascular resistance (SVR), resulting from sudden obliteration of the low-resistance ductal pathway, along with some degree of vasomotor dysregulation in the presence of maintained myocardial performance. **Consider hydrocortisone (refractory hypotension with adrenal insufficiency) with systolic and/or diastolic hypotension.

The mechanisms of PLCS are complex and incorporate both myocardial dysfunction and vascular tone dysregulation (Figure 3) (74). Ligation of the DA results in an immediate reduction of left atrial filling pressure (decrease in the preload on the left heart) as the excess volume that was being derived from the large left-to-right shunt is now eliminated. Cardiovascular compromise may initially result from the “low-preload associated reduction in contractility,” according to the Frank–Starling relationship (10, 65). Owing to the observation that the effect of preload change is greatest within the first 1–2 h after surgery and LV systolic dysfunction occurs around 8 h after surgical intervention, coinciding with clinical deterioration (2, 66), the major contributor to the hemodynamic instability in PLCS is the sudden LV exposure to the increased SVR (elevated afterload). In healthy term infants and preterm infants without an hsDA, gradual physiologic closure of the left-to-right shunt represents a return to a euvolemic state (55). Sudden removal of the left-to-right shunt following ligation, however, reflects a maladaptive low-flow state where the premature myocardium is unable to tolerate the increased afterload, leading to an increase in LV systolic wall stress, progressive LV systolic dysfunction [decreased LV shortening fraction and velocity of circumferential fiber shortening, VCFc (66)] with reduced contractility [altered stress(force)–velocity relationship (55, 66)], decline in LVO, and fall in SAP (initially with preserved or elevated diastolic BP) over a period of 6–12 h after surgery (66). The preterm myocardium is stiff and relaxes poorly; progression to diastolic dysfunction will eventually be seen in more advanced disease. Prior to proper recognition of PLCS, up to 45% of premature neonates were reported to suffer from this complication (2, 74).

Non-cardiovascular contributors to the PLCS pathophysiology, such as adrenal insufficiency, have also been investigated (67, 78, 79). Most preterm neonates undergoing PDA ligation can mount a sufficient cortisol response perioperatively and low cortisol levels do not correlate to lower CO postoperatively (67). A relatively low preoperative cortisol response (defined as post-ACTH cortisol ≤750 nmol/L) was significantly associated with postoperative hypotension (67). The nature of the hypotension in the setting of impaired adrenal responsiveness is characterized by an early (less than 4 h) fall in both systolic and diastolic arterial pressure. Low cortisol levels at 12 h postsurgery are associated with hypotension refractory to inotropes (79). Taken together, adrenal insufficiency may be an important factor in the subset of neonates with PLCS who fail to respond to catecholamines. ACTH stimulation to assess the stress response may be considered in these situations (67). Acute hypotension in the postoperative period can sometimes also arise due to surgical complications such as bleeding, tension pneumothorax, or pulmonary arterial (PA) hypertension that should also be considered in the differential diagnosis.

A holistic appraisal with careful monitoring and appropriate interpretation of all the clinical and laboratory measures of cardiovascular wellbeing, in addition to the use of targeted neonatal echocardiography (TNE), is crucial in the immediate postoperative period. Clinical and biochemical signs of CO and end organ perfusion, such as tachycardia, capillary refill time, urine output, lactate, base excess, etc., are non-specific, but may provide clues as to the state of the underlying circulation (5). Blood pressure monitoring, preferably by an invasive method, is crucial. Relying solely on MAP will be misleading as the increase in the DAP may mask the decline in MAP, as PLCS often presents with an isolated decline in SAP (1). Diastolic hypotension is unusual and should prompt searching for other causes such as hemorrhage, tension pneumothorax, sepsis, or adrenal insufficiency (2). Chest radiographs can rule out any postsurgical complications such as pneumothorax or effusion and may provide insight into the presence of pulmonary congestion secondary to left heart failure. Early TNE at 1 h post-ligation can help to identify potential cardiovascular compromise, as critically low LVO (<200 mL/kg/min) predicts the development of PLCS (68). Preoperative echocardiographic assessment of ventriculo–arterial coupling may also play a role in detecting PLCS, as infants with higher contractility (LV end-systolic elastance) in the setting of lower afterload (arterial elastance) perioperatively will not respond as favorably to acute changes in loading conditions that are evident with PDA ligation (74, 80).

The management strategy in the post PDA ligation period strives for prevention of PLCS with early modulation of the postoperative physiology of elevated afterload, reduced preload, and systolic and diastolic dysfunction (2). Baseline preoperative risk factors should be analyzed in conjunction with early echocardiographic data such as low LVO (<200 mL/kg/min) (68). If such an assessment suggests high risk of PLCS, then targeted prophylaxis with milrinone, a phosphodiesterase III inhibitor with inodilator properties, is being implemented as standard prophylactic care in the early phase when neonates are clinically stable (1). Intravenous milrinone infusion at 0.33 μg/kg/min concomitant with a bolus of 10–20 mL/kg of normal saline has been shown to significantly reduce incidence of postoperative cardiovascular instability (68). The fluid bolus augments preload and offsets the decrease in SVR associated with milrinone initiation. In institutions without access to timely postoperative echocardiography, the use of prophylactic milrinone in infants based on risk factors may be considered, but has not been properly studied (68).

A different management strategy should be considered when a neonate presents with hypotension, and echocardiographic evidence of myocardial dysfunction ± low CO in the post-ligation period. The optimal approach is to augment contractility without increasing afterload. Intravenous dobutamine represents a rational choice for patients with isolated SAP whereas epinephrine may be preferable for patients with low MAP. Agents that increase afterload such as dopamine, vasopressin, and norepinephrine should be avoided. Milrinone therapy, although biologically plausible as a preventative agent, is undesirable in an already hypotensive neonate. The role of hydrocortisone in the postoperative period is controversial. There is no evidence to support preoperative use of stress dose hydrocortisone (81). Neonates presenting with predominantly diastolic hypotension and those who have had a low or relatively low preoperative ACTH stimulation–test response may benefit from hydrocortisone.

## DA: Supporting Circulation During Maladapted Transition

There are certain states of circulatory insufficiency when the patency of the DA is desirable. The supportive role of the DA during the transitional circulation in a structurally normal heart may be best demonstrated in the setting of acute PH. Acute PH is a known cause of early cardiovascular instability and hypotension in neonates; it is characterized by a delayed fall in normal postnatal PVR, may lead to impaired RV performance, and is often associated with persistent right-to-left shunting through the DA or patent foramen ovale. The clinical consequence includes severe arterial hypoxemia and low CO. The elevated PVR diminishes PBF and pulmonary venous return leading to poor left heart filling (preload) and low CO, despite initial normal LV and RV systolic performance (82). The right-to-left shunt is an important aid to the postductal circulation, particularly in the setting of severe RV dysfunction where CO may be compromised. The initial preserved diastolic pressures with resultant systolic hypotension and right-to-left shunting of blood flow through the DA will produce differential cyanosis, detected by pre- and postductal PaO_2_ and oxygen saturation gradients. Survival in acute PH depends on proper hemodynamic coupling of the RV with the compliant PA circulation. With the failure of PVR to fall and a non-compliant PA circulation, the RV adapts to the increased afterload in acute PH by raising ventricular contractility to maintain the hemodynamic coupling of the RV to the PA circulation. However, with the RV’s limited contractile reserve and a further rise in RV afterload contributing to increased systolic wall stress, ventricular contractility will eventually not be able to meet the demand and the RV will uncouple from its afterload with a decrease in RV efficiency (83). In this setting of acute PH, removal of the placenta leads to a state of failed adaptation with increased afterload that alters the force–velocity relationship and reduces contractility and CO (55). In addition, the high RV systolic pressure in acute PH may reduce the perfusion pressure of the right coronary artery that normally is perfused in both systole and diastole further impairing RV function and CO (84). As a result, the metabolic shift from oxidative mitochondrial metabolism to aerobic glycolysis will further promote progressive RV dilation, dysfunction, failure, and possible ischemia (83).

Deterioration of RV performance may eventually affect LV function, in part due to shared myocardial fibers and ventricular–ventricular interaction (82). With advanced PH, loss of vascular tone and ventricular dysfunction will result in both diastolic and systolic hypotension. In all these scenarios, the DA must serve as an alternate pathway for RV output. When systemic pressures are less than pulmonary pressures, blood may more easily travel from the right side of the heart to the systemic circulation *via* the DA (RV–DA–Aorta route), than from the traditional pathway through the pulmonary circulation to the left side of the heart. In patients with RV dysfunction where the DA is closed or restrictive, forward blood flow is limited by increased PVR and not shunted through the DA to support the systemic circulation, leading to a further deterioration in RV performance owing to increased systolic wall stress (85). In this case, the DA can potentially be reopened with PGE infusion to allow it to serve its role in support of the RV and systemic perfusion (82). Therapeutic goals in neonates with oxygenation failure and compromised systemic hemodynamics, therefore, include reduction of PVR, improvement of ventricular–vascular compliance, and augmentation of RV (and LV) systolic function. As the DA plays an important supportive role in modulating the neonatal circulation in this maladaptive state, echocardiography provides the necessary hemodynamic information that confirms clinical suspicion and facilitates monitoring of the response to therapy in these critically ill neonates. Finally, when systemic pressures surpass pulmonary pressures with medical and/or therapeutic augmentation, the shunt direction through the DA will change from predominantly right-to-left to a left-to-right flow pattern. At that time, prostaglandin infusion can be stopped as the DA is no longer needed in its supportive capacity and it may be allowed to close.

## Role of DA in Neonates with Critical Congenital Cardiac Defects

There are certain critical congenital cardiac lesions that can lead to acute cardiovascular collapse and rely on the supportive role of the DA to provide systemic and/or PBF (Table 1). Neonates with congenital heart defects (CHD) and ductal dependency can be categorized into three broad groups: (1) right-sided obstructive lesions with DA dependent pulmonary circulation in the setting of severe restriction of PBF, (2) left-sided obstructive lesions with DA-dependent systemic circulation; and (3) adequate mixing of the pulmonary and systolic blood flow that is DA dependent to maintain the circulation in series.

**Table 1:** DA in specific critical congenital cardiac defects.

Role of DA	Examples	Clinical findings
DA required for adequate pulmonary blood flow (PBF)	Tetralogy of Fallot depending on degree of pulmonary stenosis Double-outlet right ventricle with subaortic ventricular septal defect (VSD) and pulmonary stenosis Tricuspid atresia Pulmonary atresia Critical pulmonary stenosis Severe Ebstein’s anomaly Single ventricle with pulmonary stenosis	Infant presents with cyanosis and hypoxia Inadequate PBF initially manifests with systolic hypotension due to compromised left ventricle preload followed by diastolic (combined) hypotension

DA required for adequate systemic blood flow (SBF)	Aortic stenosis Coarctation of the aorta Aortic arch interruption Hypoplastic left heart syndrome Multiple left heart defects	Infant presents with signs of poor perfusion with weak or absent pulses in lower extremities Inadequate SBF manifests with profound systolic hypotension and may progress to shock

DA required for right to left shunt to ensure adequate atrial level mixing in parallel circulations with poor mixing	d-transposition of great arteries d-transposition of great arteries and VSD Double-outlet right ventricle with sub-pulmonary VSD	Infant may present with early profound hypoxia in presence of restrictive PFO Parallel circulations without mixing ultimately results in profound, lethal systemic hypoxia

DA may contribute to increased PBF and cyanosis in lesions with complete mixing	Total anomalous pulmonary venous connection Truncus arteriosus Single ventricle without pulmonary stenosis Double-outlet right ventricle with sub-aortic VSD and without significant pulmonic stenosis	Infant usually presents with mild hypoxia Infants with obstructed pulmonary venous connection will present with profound systemic hypoxia and systolic hypotension

*DA, ductus arteriosus*.

For those infants diagnosed in the antenatal period with ductal-dependent CHD, PGE is initiated shortly after delivery (86). In neonates diagnosed postnatally with ductal-dependent CHD, PGE must be initiated as soon as possible, sometimes at a higher dose to facilitate reopening of the DA. The specific role of the DA is dependent on the type of CHD diagnosed in each patient. In congenital heart lesions that involve an anatomic obstruction to PBF infants present as cyanotic and hypoxic with usual oxygen saturations between 75 and 85%. In the absence of a patent DA, infants will become profoundly hypoxic with little to no source of PBF as the DA constricts and closes. This may further manifest as early systolic hypotension due to the severely compromised LV preload, followed by diastolic hypotension, as the hypoxemia compromises vascular tone. Further complications may include arrhythmias, respiratory failure, and heart failure. The DA is required until surgical intervention or palliation to provide an alternate source of PBF.

In congenital heart lesions that involve an anatomic obstruction to SBF, the DA is required to maintain adequate systemic perfusion (87). These lesions are termed ductal-dependent for SBF and present with signs of poor perfusion in the presence of a constricting or closed DA with profound systolic hypotension. Additional signs and symptoms include weak or absent pulses, metabolic acidosis, lactic acidosis, and shock. The purpose of the DA in these cases is to facilitate right-to-left shunting of blood, albeit with a lower oxygen concentration, which bypasses the varying degrees of obstructive left-sided lesion. PGE is required to maintain patency of the DA with permissive hypoxemia until surgical repair is performed.

The DA has a specific role in lesions with parallel circulations and poor mixing (87). With the circulations in parallel, atrial level communication is critical for mixing. The DA provides right-to-left shunting in order to increase left atrial pressures and improve atrial level mixing. The DA must remain patent until adequate atrial mixing is established and confirmed; this may require atrial septostomy in the newborn period. If adequate atrial mixing is maintained, the DA may be allowed to close prior to surgical correction of the underlying pathology.

The role of the DA is more complicated in other mixing lesions, though the clinical presentation may still be cyanosis. In lesions with complete mixing in the presence of a left-to-right shunt at the DA, the source of cyanosis is often from increased PBF, which can lead to tachypnea and respiratory distress (88). In these cases, supplemental oxygen and other interventions that decrease PVR can contribute to a further increase in PBF at the expense of the systemic circulation, ultimately resulting in hypotension and shock. PGE is often avoided to minimize worsening systemic perfusion that would be associated with a significant left to right shunt at the DA. In cases where ductal-dependent congenital lesions accompany a mixing lesion, such as coarctation of the aorta or interrupted aortic arch, PGE will be required to maintain DA patency in support of the accompanying lesions.

## Conclusion

The DA is essential in fetal life, important in early transition, potentially detrimental in select preterm populations, and supportive in specific disease processes in the structurally normal and abnormal heart. Cardiovascular compromise and hypotension may occur at each postnatal stage and the DA plays a dynamic role in modulating the neonatal circulation. Therefore, understanding the pathophysiologic basis of such instability arising due to the DA and early recognition of the typical clinical phenotype characterizing the various disease stages is essential for proper diagnosis, monitoring, and therapeutic support. In certain cases, the treatment for an hsDA can lead to serious cardiovascular compromise in premature neonates. Analyzing systolic and diastolic hypotension separately, instead of focusing solely on MAP, may provide early insight into the mechanistic contribution of the DA, which can then be evaluated in conjunction with using TNE. A holistic diagnostic approach that synthesizes clinical signs, biochemical values, and echocardiographic evidence can be utilized to devise focused, individualized, physiology-based treatment strategies that optimize management of these challenging patients and has the potential to improve patient outcomes.

## Author Contributions

DR and SB drafted the first version and equally contributed to writing the first draft of the review. PL and PM are responsible for study conception and performed the critical revision. Each author has seen and provided final approval of the version to be published, with agreement to be accountable for all aspects of the work.

## Conflict of Interest Statement

The authors declare that the research was conducted in the absence of any commercial or financial relationships that could be construed as a potential conflict of interest.

## Abbreviations

CO, cardiac output; DA, ductus arteriosus; DAP, diastolic arterial pressure; LVO, left ventricle output; MAP, mean arterial pressure; PDA, patent ductus arteriosus; PVR, pulmonary vascular resistance; PBF, pulmonary blood flow; SBF, systemic blood flow; SAP, systolic arterial pressure; SV, stroke volume; SVR, systemic vascular resistance.

## References

[1] El-Khuffash AF Weisz A McNamara PJ Reflections of the changes in patent ductus arteriosus management during the last 10 years. Arch Dis Child Fetal Neonatal Ed (2016) 5:F474–8. 10.1136/archdischild-2014-306214 27118761

[2] EL-Khuffash AF Jain A McNamara PJ Ligation of the patent ductus arteriosus in preterm infants: understanding the physiology. J Pediatr (2013) 6:1100–6. 10.1016/j.jpeds.2012.12.094 23410600

[3] Laudy JA Wladimiroff JW. The fetal lung 1: developmental aspects. Ultrasound Obstet Gynecol (2000) 16:284–90. 10.1046/j.1469-0705.2000.00228.x 11169299

[4] Prsa M Sun L van Amerom J Yoo SJ Grosse-Wortmann L Jaeggi E Reference ranges of blood flow in the major vessels of the normal human fetal circulation at term by phase-contrast magnetic resonance imaging. Circ Cardiovasc Imaging (2014) 7:663–70. 10.1161/circimaging.113.001859 24874055

[5] El-Khuffash AF McNamara PJ. Hemodynamic assessment and monitoring of premature infants. Clin Perinatol (2017) 44:377–93. 10.1016/j.clp.2017.02.001 28477667

[6] Rudolph AM, editor. The ductus arteriosus and persistent patency of the ductus arteriosus. 3rd ed Congenital Diseases of the Heart. Chichester, UK: Wiley-Blackwell (2009). p. 115–47.

[7] Gournay VR. The ductus arteriosus: physiology, regulation, and functional and congenital anomalies. Arch Cardiovasc Dis (2011) 104:578–85. 10.1016/j.acvd.2010.06.006 22117910

[8] Gao Y Raj JU. Regulation of the pulmonary circulation in the fetus and newborn. Physiol Rev (2010) 90:1291–335. 10.1152/physrev.00032.2009 20959617

[9] Hillman NH Kallapur SG Jobe AH. Physiology of transition from intrauterine to extrauterine life. Clin Perinatol (2012) 39:769–83. 10.1016/j.clp.2012.09.009 23164177 PMC3504352

[10] Giesinger RE McNamara PJ Hemodynamic instability in the critically ill neonat: an approach to cardiovascular support based on disease pathophysiology. Semin Perinatol (2016) 40:174–88. 10.1053/j.semperi.2015.12.005 26778235

[11] Gittenberger-de Groot AC Strengers JLM Mentink M Poelmann RE Patterson DF. Histologic studies on normal and persistent ductus arteriosus in the dog. J Am Coll Cardiol (1985) 6:394–404. 10.1016/S0735-1097(85)80178-9 4019926

[12] Quinn D Cooper B Clyman RI. Factors associated with permanent closure of the ductus arteriosus: a role for prolonged indomethacin therapy. Pediatrics (2002) 110:e10–10. 10.1542/peds.110.1.e10 12093991

[13] Dempsey EM Barrington KJ. Diagnostic criteria and therapeutic interventions for the hypotensive very low birth weight infant. J Perinatol (2006) 26:677–81. 10.1038/sj.jp.7211579 16929346

[14] Pellicer A Valverde E Elorza MF Madero R Gaya F Quero J Cardiovascular support for low birth weight infants and cerebral hemodynamics: a randomized, blinded, clinical trial. Pediatrics (2005) 115:1501–12. 10.1542/peds.2004-1396 15930210

[15] British Association of Perinatal Medicine and the Research Unit of the Royal College of Physicians. Development of audit measures and guidelines for good practice in the management of neonatal respiratory distress syndrome. Arch Dis Child (1992) 67(10 Spec No):1221–7. 10.1136/adc.67.10_Spec_No.1221 1444567 PMC1590463

[16] Watkins AM Wes CR Cooke RW. Blood pressure and cerebral haemorrhage and ischaemia in very low birthweight infants. Early Hum Dev (1989) 19:103–10. 10.1016/0378-3782(89)90120-5 2737101

[17] Munro MJ Walker AM Barfield CP. Hypotensive extremely low birth weight infants have reduced cerebral blood flow. Pediatrics (2004) 114:1591–6. 10.1542/peds.2004-1073 15574619

[18] Borch K Lou HC Greisen G. Cerebral white matter blood flow and arterial blood pressure in preterm infants. Acta Paediatr (2010) 99:1489–92. 10.1111/j.1651-2227.2010.01856.x 20456278 PMC3068289

[19] Nuntnarumit P Yang W Bada-Ellzey HS Blood pressure measurements in the newborn. Clin Perinatol (1999) 26:981–96. 10572732

[20] Hegyi T Anwar M Carbone MT Ostfeld B Hiatt M Koons A Blood pressure ranges in premature infants: II. The first week of life. Pediatrics (1996) 97:336–42. 8604266

[21] Hegyi T Carbone MT Anwar M Ostfeld B Hiatt M Koons A The first hours of life. J Pediatr (1994) 124:627–33. 10.1016/S0022-3476(05)83146-4 8151481

[22] Batton B Batton D Riggs T. Blood pressure during the first 7 days in premature infants born at postmenstrual age 23 to 25 weeks. Am J Perinatol (2007) 2:107–15. 10.1055/s-2007-970178 17304424

[23] Kusaka T Okubo K Nagano K Isobe K Itoh S. Cerebral distribution of cardiac output in newborn infants. Arch Dis Child Fetal Neonatal Ed (2005) 90:F77–8. 10.1136/adc.2004.058487 15613584 PMC1721835

[24] Ashwal A Dale PS Longo LD. Regional cerebral blood flow: studies in the fetal lamb during hypoxia, hypercapnia, acidosis, and hypotension. Pediatr Res (1984) 18:1309–16. 10.1203/00006450-198412000-00018 6441142

[25] Osborn DA Evans N Kluckow M. Clinical detection of low upper body blood flow in very premature infants using blood pressure, capillary refill time, and central-peripheral temperature difference. Arch Dis Child Fetal Neonatal Ed (2004) 89:F168–73. 10.1136/adc.2002.023796 14977905 PMC1756033

[26] Kluckow M. Low systemic blood flow and pathophysiology of the preterm transitional circulation. Early Hum Dev (2005) 81:429–37. 10.1016/j.earlhumdev.2005.03.006 15935920

[27] Kluckow M Evans N. Low superior vena cava flow and intraventricular haemorrhage in preterm infants. Arch Dis Child Fetal Neonatal Ed (2000) 82:F188–94. 10.1136/fn.82.3.F188 10794784 PMC1721081

[28] Groves AM Kuschel CA Knight DB Skinner JR Relationship between blood pressure and blood flow in newborn preterm infants. Arch Dis Child Fetal Neonatal Ed (2008) 92:F29–32. 10.1136/adc.2006.109520 17475696

[29] Sarkar S Dechert R Schumacher RE Donn SM. Is refractory hypotension in preterm infants a manifestation of early ductal shunting? J Perinatol (2007) 27:353–8. 10.1038/sj.jp.7211749 17443200

[30] Havranek T Rahimi M Hall H Armbrecht E. Feeding preterm neonates with patent ductus arteriosus (PDA): intestinal blood flow characteristics and clinical outcomes. J Matern Fetal Neonatal Med (2015) 28:526–30. 10.3109/14767058.2014.923395 24824108

[31] Shimada S Kasai T Konishi M Fujiwara T. Effects of patent ductus arteriosus on left ventricular output and organ blood flows in preterm infants with respiratory distress syndrome treated with surfactant. J Pediatr (1994) 125:270–7. 10.1016/S0022-3476(94)70210-1 8040777

[32] Clyman RI Mauray F Heymann MA Roman C. Cardiovascular effects of patent ductus arteriosus in preterm lambs with respiratory distress. J Pediatr (1987) 111:579–87. 10.1016/S0022-3476(87)80126-9 3655990

[33] Rowland DG Gutgesell HP. Noninvasive assessment of myocardial contractility, preload, and afterload in healthy newborn infants. Am J Cardiol (1995) 75:818–21. 10.1016/S0002-9149(99)80419-6 7717287

[34] Crystal MA Yacouby S Petit CJ. Ischemic changes associated with a large patent arterial duct in small infants. Catheter Cardiovasc Interv (2014) 83:95–8. 10.1002/ccd.24639 22936526

[35] Fluri S Pavlovic M Wagner BP. Patent arterial duct, bottle-meal, and fatal myocardial ischaemia. Cardiol Young (2010) 20:108–10. 10.1017/S1047951109991028 20188018

[36] Marijianowski MM van der Loos CM Mohrschladt MF Becker AE. The neonatal heart has a relatively high content of total collagen and type I collagen, a condition that may explain the less compliant state. J Am Coll Cardiol (1994) 23:1204–8. 10.1016/0735-1097(94)90612-2 8144790

[37] Wong SN Lo RN Hui PW. Abnormal renal and splanchnic arterial Doppler pattern in premature babies with symptomatic patent ductus arteriosus. J Ultrasound Med (1990) 9:125–30. 10.7863/jum.1990.9.3.125 2407859

[38] Coombs RC Morgan ME Durbin GM Booth IW McNeish AS. Gut blood flow velocities in the newborn: effects of patent ductus arteriosus and parenteral indomethacin. Arch Dis Child (1990) 65:1067–71. 10.1136/adc.65.10_Spec_No.1067 2241229 PMC1590251

[39] McCurnin D Clyman RI. Effects of a patent ductus arteriosus on postprandial mesenteric perfusion in premature baboons. Pediatrics (2008) 122:e1262–7. 10.1542/peds.2008-2045 19001037 PMC2597012

[40] McCurnin D Seidner S Chang LY Waleh N Ikegami M Petershack J Ibuprofen-induced patent ductus arteriosus closure: physiologic, histologic, and biochemical effects on the premature lung. Pediatrics (2008) 121:945–56. 10.1542/peds.2007-2051 18450898 PMC11790498

[41] Lemmers PM Benders MJ D’Ascenzo R Zethof J Alderliesten T Kersbergen KJ Patent ductus arteriosus and brain volume. Pediatrics (2016) 137(4):e20153090. 10.1542/peds.2015-3090 27030421

[42] Evans N Kluckow M. Early ductal shunting and intraventricular haemorrhage in ventilated preterm infants. Arch Dis Child Fetal Neonatal Ed (1996) 75:F183–6. 10.1136/fn.75.3.F183 8976684 PMC1061197

[43] Noori S Seri I. Hemodynamic antecedents of peri/intraventricular hemorrhage in very preterm neonates. Semin Fetal Neonatal Med (2015) 20:232–7. 10.1016/j.siny.2015.02.004 25818879

[44] Fink D El-Khuffash AF McNamara PJ Nitzan I Hammerman C. Tale of two patent ductus arteriosus severity scores: similarities and differences. Am J Perinatol (2018) 35:55–8. 10.1055/s-0037-1605576 28787748

[45] El-Khuffash A James AT Corcoran JD Dicker P Franklin O Elsayed YN A patent ductus arteriosus severity score predicts chronic lung disease or death before discharge. J Pediatr (2015) 167:1354–61. 10.1016/j.jpeds.2015.09.028 26474706

[46] Kluckow M Evans N. Early echocardiographic prediction of symptomatic patent ductus arteriosus in preterm infants undergoing mechanical ventilation. J Pediatr (1995) 127:774–9. 10.1016/S0022-3476(95)70172-9 7472835

[47] Sehgal A Paul E Menahem S. Functional echocardiography in staging for ductal disease severity: role in predicting outcomes. Eur J Pediatr (2013) 172:179–84. 10.1007/s00431-012-1851-0 23052621

[48] Schena F Francescato G Cappelleri A Picciolli I Mayer A Mosca F Association between hemodynamically significant patent ductus arteriosus and bronchopulmonary dysplasia. J Pediatr (2015) 166:1488–92. 10.1016/j.jpeds.2015.03.012 25882876

[49] El Hajjar M Vaksmann G Rakza T Kongolo G Storme L. Severity of the ductal shunt: a comparison of different markers. Arch Dis Child Fetal Neonatal Ed (2005) 90:F419–22. 10.1136/adc.2003.027698 16113155 PMC1721944

[50] Dempsey EM El-Khuffash AF Objective cardiovascular assessment in the neonatal intensive care unit. Arch Dis Child Fetal Neonatal Ed (2018) 103:F72–7. 10.1136/archdischild-2017-313837 29127152

[51] Azhibekov T Noori T Soleymani S Seri I. Transitional cardiovascular physiology and comprehensive hemodynamic monitoring in the neonate: relevance to research and clinical care. Semin Fetal Neonatal Med (2014) 19:45–53. 10.1016/j.siny.2013.09.009 24555196

[52] Azhibekov T Soleymani S Lee BH Noori S Seri I. Hemodynamic monitoring of the critically ill neonate: an eye on the future. Semin Fetal Neonatal Med (2015) 20:246–54. 10.1016/j.siny.2015.03.003 25841985

[53] Seri I Noori S. Diagnosis and treatment of neonatal hypotension outside the transitional period. Early Hum Dev (2005) 81:405–11. 10.1016/j.earlhumdev.2005.03.008 15882935

[54] Fajardo MF Claure N Swaminathan S Sattar S Vasquez A D’Ugard C Effect of positive end-expiratory pressure on ductal shunting and systemic blood flow in preterm infants with patent ductus arteriosus. Neonatology (2014) 105:9–13. 10.1159/000355146 24193163

[55] Giesinger RE Bailey LJ Deshpande P McNamara PJ Hypoxic-ischemic encephalopathy and therapeutic hypothermia: the hemodynamic perspective. J Pediatr (2016) 180:22–30.e2. 10.1016/j.jpeds.2016.09.009 27742125

[56] Liet JM Boscher C Gras-Leguen C Gournay V Debillon T Roze JC. Dopamine effects on pulmonary artery pressure in hypotensive preterm infants with patent ductus arteriosus. J Pediatr (2002) 140:373–5. 10.1067/mpd.2002.123100 11953739

[57] Jaillard S Houfflin-Debarge V Riou Y Rakza T Klosowski S Lequien P Effects of catecholamines on the pulmonary circulation in the ovine fetus. Am J Physiol Regul Integr Comp Physiol (2001) 281:R607–14. 10.1152/ajpregu.2001.281.2.R607 11448866

[58] Seri I Cardiovascular, renal, and endocrine actions of dopamine in neonates and children. J Pediatr (1995) 126:333–44. 10.1016/S0022-3476(95)70445-0 7869189

[59] Zhang J Penny DJ Kim NS Yu VY Smolich JJ. Mechanisms of blood pressure increase induced by dopamine in hypotensive preterm neonates. Arch Dis Child Fetal Neonatal Ed (1999) 81:F99–104. 10.1136/fn.81.2.F99 10448176 PMC1720986

[60] Pezzati M Vangi V Biagiotti R Bertini G Cianciulli D Rubaltelli FF. Effects of indomethacin and ibuprofen on mesenteric and renal blood flow in preterm infants with patent ductus arteriosus. J Pediatr (1999) 135:733–8. 10.1016/S0022-3476(99)70093-4 10586177

[61] Sehgal A Ramsden CA McNamara PJ. Indomethacin impairs coronary perfusion in infants with hemodynamically significant ductus arteriosus. Neonatology (2012) 101:20–7. 10.1159/000327844 21791936

[62] Van Bel F Van de Bor M Stijnen T Baan J Ruys JH. Cerebral blood flow velocity changes in preterm infants after a single dose of indomethacin: duration of its effect. Pediatrics (1989) 84:802–7. 2677960

[63] El-Mashad AE El-Mahdy H El Amrousy D Elgendy M. Comparative study of the efficacy and safety of paracetamol, ibuprofen, and indomethacin in closure of patent ductus arteriosus in preterm neonates. Eur J Pediatr (2017) 176:233–40. 10.1007/s00431-016-2830-7 28004188

[64] Levy PT El-Khuffash AF Pulmonary arterial hypertension after ibuprofen treatment in the first week of life? J Pediatr (2017) 182:408–9. 10.1016/j.jpeds.2016.11.047 27939126

[65] Noori S Friedlich P Seri I Wong P Changes in myocardial function and hemodynamics after ligation of the ductus arteriosus in preterm infants. J Pediatr (2007) 6:597–602. 10.1016/j.jpeds.2007.01.035 17517241

[66] McNamara PJ Stewart L Shivananda SP Stephens D Sehgal A. Patent ductus arteriosus ligation is associated with impaired left ventricular systolic performance in premature infants weighing less than 1000 g. J Thorac Cardiovasc Surg (2010) 140:944. 10.1016/j.jtcvs.2010.01.011 20363478

[67] El-Khuffash AF McNamara PJ Lapointe A Jain A. Adrenal function in preterm infants undergoing patent ductus arteriosus ligation. Neonatology (2013) 104:28–33. 10.1159/000350017 23635520

[68] Jain A Sahni M El-Khuffash A Khadawardi E Sehgal A McNamara PJ. Use of targeted neonatal echocardiography to prevent postoperative cardiorespiratory instability after patent ductus arteriosus ligation. J Pediatr (2012) 160:584–9.e1. 10.1016/j.jpeds.2011.09.027 22050874

[69] Teixeira LS Shivananda SP Stephens D Van Arsdell G McNamara PJ. Postoperative cardiorespiratory instability following ligation of the preterm ductus arteriosus is related to early need for intervention. J Perinatol (2008) 28:803–10. 10.1038/jp.2008.101 18615091

[70] Moin F Kennedy KA Moya FR. Risk factors predicting vasopressor use after patent ductus arteriosus ligation. Am J Perinatol (2003) 20:313–20. 10.1055/s-2003-42693 14528401

[71] Natarajan G Chawla S Aggarwal S. Short-term outcomes of patent ductus arteriosus ligation in preterm neonates: reason for concern? Am J Perinatol (2010) 27:431–7. 10.1055/s-0029-1243367 20013575

[72] Naik-Mathuria B Chang S Fitch ME Westhoff J Brandt ML Ayres NA Patent ductus arteriosus ligation in neonates: preoperative predictors of poor postoperative outcomes. J Pediatr Surg (2008) 43:1100–5. 10.1016/j.jpedsurg.2008.02.037 18558190

[73] Zahn EM Peck D Philip A Nevin P Basaker K Simmons C Transcatheter closure of patent ductus arteriosus in extremely premature newborns: early results and midterm follow-up. J Am Coll Cardiol Intv (2016) 9:2429–37. 10.1016/j.jcin.2016.09.019

[74] Noori S Kumar R Pre-dicting post-ligation syndrome. J Thorac Cardiovasc Surg (2017) 154:2060–1. 10.1016/j.jtcvs.2017.08.048 28967417

[75] Marshall TA Marshall F Reddy PP. Physiologic changes associated with ligation of the ductus arteriosus in preterm infants. J Pediatr (1982) 101:749–53. 10.1016/S0022-3476(82)80311-9 7131153

[76] Petit A Morelon P Massin F Diebold H Brenot R Louis P Systemic arterial hypertension caused by discharge of catecholamines following ligation of ductus arteriosus. Arch Mal Coeur Vaiss (1984) 77:586–9. 6428358

[77] Davierwala P Thakur N Babu P Reddy S Kumar P Menon R. Unexplained systemic hypertension after closure of ductus arteriosus. Asian Cardiovasc Thorac Ann (2002) 10:78–9. 10.1177/021849230201000123 12079982

[78] Noori S McNamara P Jain A Lavoie PM Wickremasinghe A Merritt TA Catecholamine-resistant hypotension and myocardial performance following patent ductus arteriosus ligation. J Perinatol (2015) 35:123–7. 10.1038/jp.2014.15 25118721 PMC4310792

[79] Clyman RI Wickremasinghe A Merritt TA Solomon T McNamara PJ Jain A Hypotension following patent ductus arteriosus ligation: the role of adrenal hormones. J Pediatr (2014) 164:1449–55.e1. 10.1016/j.jpeds.2014.01.058 24636853 PMC4035426

[80] Gray MA Graham EM Atz AM Bradley SM Kavarana MN Chowdhury S. Preoperative echocardiographic measures of left ventricular mechanics are associated with postoperative vasoactive support in preterm infants undergoing patent ductus arteriosus ligation. J Thorac Cardiovasc Surg (2017) 154:2054–9.e1. 10.1016/j.jtcvs.2017.06.051 28743382 PMC5685891

[81] Satpute MD Donohue PK Vricella L Aucott SW Cardiovascular instability after patent ductus arteriosus ligation in preterm infants: the role of hydrocortisone. J Perinatol (2012) 32:685–9. 10.1038/jp.2011.166 22094490

[82] Jain A McNamara PJ. Persistent pulmonary hypertension of the newborn: advances in diagnosis and treatment. Semin Fetal Neonatal Med (2015) 20:262–71. 10.1016/j.siny.2015.03.001 25843770

[83] Ryan JJ Huston J Kutty S Hatton ND Bowman L Tian L Right ventricular adaptation and failure in pulmonary arterial hypertension. Can J Cardiol (2015) 31:391–406. 10.1016/j.cjca.2015.01.023 25840092 PMC4385216

[84] Bogaard HJ Abe K Vonk Noordegraaf A Voelkel NF. The right ventricle under pressure: cellular and molecular mechanisms of right-heart failure in pulmonary hypertension. Chest (2009) 135:794–804. 10.1378/chest.08-0492 19265089

[85] Mohseni-Bod H Bohn D. Pulmonary hypertension in congenital diaphragmatic hernia. Semin Pediatr Surg (2007) 16:126–33. 10.1053/j.sempedsurg.2007.01.008 17462565

[86] Neutze JM Starling MD Elliott RB Barratt-Boyes BG. Palliation of cyanotic congenital heart disease in infancy with E-type prostaglandins. Circulation (1977) 55:238–41. 10.1161/01.CIR.55.2.238 64317

[87] Friedman AH Fahey JT. The transition from fetal to neonatal circulation: normal responses and implications for infants with heart disease. Semin Perinatol (1993) 17:106–21. 8327901

[88] Haworth SG Bull C Physiology of congenital heart disease. Arch Dis Child (1993) 68:707–11. 10.1136/adc.68.5.707 8323346 PMC1029349

